# Individual factors determine landing impacts in rested and fatigued cheerleaders

**DOI:** 10.3389/fspor.2024.1419783

**Published:** 2024-08-13

**Authors:** Andreas Müller, Robert Rockenfeller, Ameet K. Aiyangar

**Affiliations:** ^1^ Institute for Medical Engineering and Information Processing (MTI Mittelrhein), University of Koblenz, Koblenz, Germany; ^2^ Mechanical Systems Engineering, Swiss Federal Laboratories for Materials Science and Technology (EMPA), Duebendorf, Switzerland; ^3^ Institute of Sports Science, University of Koblenz, Koblenz, Germany; ^4^ Mathematical Institute, University of Koblenz, Koblenz, Germany; ^5^ School of Science, Technology and Engineering, University of the Sunshine Coast, Sippy Downs, QLD, Australia; ^6^ School of Biomedical Sciences, University of Queensland, St Lucia, QLD, Australia; ^7^ Department of Orthopedic Surgery, University of Pittsburgh, Pittsburgh, PA, United States; ^8^ Faculty of Engineering and Sciences, University of Adolfo Ibanez, Vina del Mar, Chile; ^9^ Faculty of Medicine, University of Bern, Bern, Switzerland

**Keywords:** cheerleading, short recovery stress scale, Borg scale, statistical parametric mapping, ANOVA, force plate

## Abstract

High vertical ground reaction forces (VGRF) during landings following acrobatic elements in artistic gymnastics is associated with trunk and lower extremity injury risk. As similar data regarding injury risk factors in cheerleading are scarce, the purpose of this study was to assess VGRF in pop-off dismounts of rested and fatigued flyers in cheerleaders. Fifteen German cheerleaders were recruited for this study, including seven female flyers and eight male bases. It was expected that performance would change in fatiguing athletes, potentially increasing the risk for injuries. However, neither the mean VGRF (rested: 6.0±1.9 BW, fatigued: 6.2±1.3 BW, overall range: 2.1–14.9 BW) nor the individual VGRF-time courses of the flyers changed significantly after the workout. Instead, we show that the flyers’ ability to land – but not the bases’ ability to catch – significantly influences the maximum and time-resolved impacts.

## 1 Introduction

The International Olympic Committee (IOC) acknowledged the International Cheer Union (ICU) as the world governing body of cheerleading in July 2021, with the prospect of eventually becoming an Olympic sport. Contrary to the US, cheerleading is a fringe sport in Germany, yet growing in numbers. In recent years, a league system has been developed under the aegis of the “German Cheerleading and Cheerperformance Association” (Cheerleading und Cheerperformance Verband Deutschland, CCVD). During competitive championships, teams consisting of throwing (*bases*) and thrown members (*flyers*) perform routines with diverse elements – e.g. *(partner)stunts*, *pyramids*, or *baskets* (1, 2) – in order to obtain points by the judges. The permitted difficulty of stunts increases with athlete level (from 0 for beginners to 7 for professionals). The more complex and well-performed the routine, the more points for the team, particularly if the flyer does not *drop* (fall) (1). Hence, particular attention is directed to the smooth dismount of the flyer from great heights during training; more precisely, from one and a half body heights in partnerstunts and up to two and a half body heights in pyramids (1).

Landing from such great heights constitutes a huge injury risk factor for the flyers (3). In an attempt to mitigate these injury risks, the CCVD provides training guidelines to ensure a safe and preferably injury-free training practice (4). However, as cheerleading is a fringe sport in Germany, there is limited information related to frequency, severity and prevention of injuries (5). The same holds true for whole Europe: A PubMed search with keywords “Cheerleading and injuries” yielded 96 results of which 63 explicitly contained “US or USA” but none contained “Germany or Europe” (search results as of December 2023). Most injuries in US cheerleaders are strains and sprains in the ankle and lower extremities (3, 6–9). Approximately 17% of cheerleaders [193 out of 1,115, (10), Table 4] exhibited fractures, including stress fractures. The most catastrophic injuries occurred after landing on hard floors (11).

By the same token, injuries are predominantly linked to landings in floor exercises or dismounts in gymnastics (12–14) – a related sport. Hume et al. (15) further emphasize that uncontrolled and repetitive landings can lead to both acute and overuse injuries. It is hence unsurprising that the *Code of Points* for women’s gymnastics stipulates a controlled landing on two feet for floor exercises (16, 17). According to Bradshaw and Hume (16), any modifications to the Code of Points should be made with athlete safety in mind, which in turn is linked to biomechanical considerations.

High impact forces during landing from great height are considered to be one of the leading biomechanical causes for injuries in athletes across disciplines (16, 18–20). Although some cheerleading-related injuries are known to occur during gymnastics parts, most happen during stunts (3, 21). These impact forces are commonly quantified by measuring the *vertical ground reaction forces* (VGRF) via force plates in various sports such as running, basketball, tennis, football, volleyball, skiing or gymnastics (20, 22). Depending on the injury risk assessment, one may be interested in peak VGRF, mean VGRF, or cumulative VGRF (impulse). For this study, we focus mainly on peak VGRF, i.e. the maximum recorded impact force over a certain time interval of interest. Peak VGRF during landing can range from four times the body weight (4 BW) after a 0.32 m jump to 11 BW after a 1.28 m jump (19). Hume et al. (15) reported VGRF of two-foot landings in gymnastics from 5 BW in training to 11 BW in competitions, and even up to 18 BW during unusual foot placement. To our knowledge, no measurements of VGRF during cheerleading stunts exist, where the flyers’ landings are supported by their bases. Here, we present preliminary VGRF measurements after dismounting from a particular stunt, the so-called *pop-off*, where flyers drop-land on the ground after jumping straight from the outstretched arms of their bases. As studies suggest an association between athletes’ fatigue and injury risk [see citations in Bagnulo (6)], stunts were performed by flyer-base pairs both in a rested state and after high-intensity workouts to measure the effect of fatigued stunt partners. Additionally, counter-movement jumps (CMJ) were utilized to measure the VGRF impacts of individual athletes to assess both their landing mechanics and their fatigue (23).

We aimed to address the following: (1) does a fatiguing workout alter the landing characteristics of the flyer or the catching ability of the base (2) is the landing impact determined predominantly by the base or the flyer (3) is there an association between two-foot landing impacts during CMJ and pop-off stunts, and, finally (4) monitor the effect of a fatiguing workout on VGRF landing profiles.

## 2 Method

### 2.1 Participants

We invited 15 level-6 athletes from a German coed team, with three to eleven years’ experience in cheerleading. All athletes had already competed at national championships and had given their written informed consent to participate in this study.

The requirement for an approval was waived by the local ethics committee within the University of Koblenz. The seven flyers – denoted as F1,…,F7 – were exclusively female with a mean age of 24.6±2.5 years (mean ± standard deviation). The eight bases – denoted as B1,…,B8 – were exclusively male with a mean age of 30.1±4.2 years). Table 1 further summarizes data on the participants, such as age, mass, height, and performed pop-offs on each test day (see below). Mass and height were measured on site by the experimenters before the trials, using the force plate and measuring tape.

**Table 1:** Participants data sheet.

Role	Person	Sex	Age	Mass	Height	Pop-offs performed at		
	(identifier)		(years)	(kg)	(cm)	Day 1	Day 2	Day 3
Flyer	F1	female	25	54.1	155	–	–	13 {25}
	F2	female	27	52.2	163	–	–	32 {42}
	F3	female	25	48.5	159	–	36 {43}	–
	F4	female	21	58.0	169	–	–	14 {16}
	F5	female	25	57.9	158	8 {13}	–	–
	F6	female	21	54.0	167	11 {13}	–	16 {20}
	F7	female	28	46.3	167	–	–	21 {25}
All flyers			24.6	53.0	162.6	19 {26}	36 {43}	96 {128}
			± 2.5	± 4.1	± 5.0			
Base	B1	male	35	120.3	193	–	–	14 {16}
	B2	male	30	86.5	181	–	–	12 {17}
	B3	male	27	92.0	175	8 {13}	12 {13}	9 {16}
	B4	male	26	76.5	185	–	–	10 {16}
	B5	male	25	83.5	190	11 {13}	11 {17}	10 {16}
	B6	male	38	82.6	181	–	–	12 {16}
	B7	male	30	113.7	178.5	–	–	16 {16}
	B8	male	30	81.6	172	–	13 {13}	13 {15}
All bases			30.1	92.1	181.9	19 {26}	36 {43}	96 {128}
			± 4.2	± 15.0	± 6.7			
All athletes			27.5	73.8	172.9	Successful pop-offs: 151		
			± 4.5	± 22.6	± 11.3	Total pop-offs: {197}		

Pseudonymization of the seven female flyers (F1,…,F7) and eight male bases (B1,…,B8) together with their age, mass, and height. Mean values for flyers, bases, and all athletes are given alongside their standard deviation (± sign). Last three columns contain the number of successful and total {in curly brackets} pop-offs, respectively, for each athlete at the test days.

### 2.2 Technical description of the pop-off stunt

**Figure 1:**
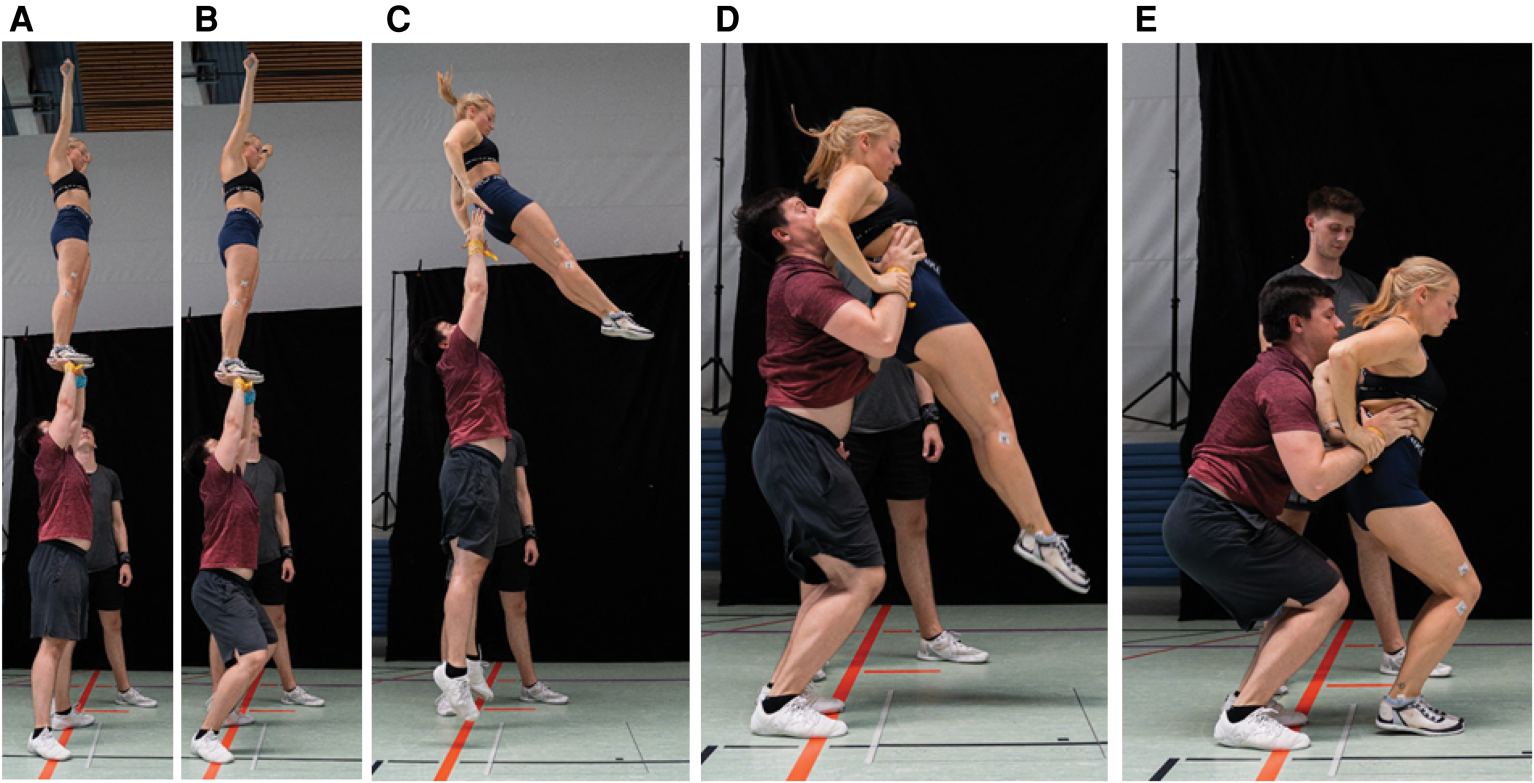
Pop-off technique in five sequences. (**A**) The flyer is in the extended position. (**B**) The base bends the knees to initiate the dismount and to signal the flyer. (**C**)–(**D**) the base grabs the hips and tries to decelerate the flyer, while the flyer grabs the wrists of the base. (**E**) The flyer lands on the force plate.

**Figure 2:**
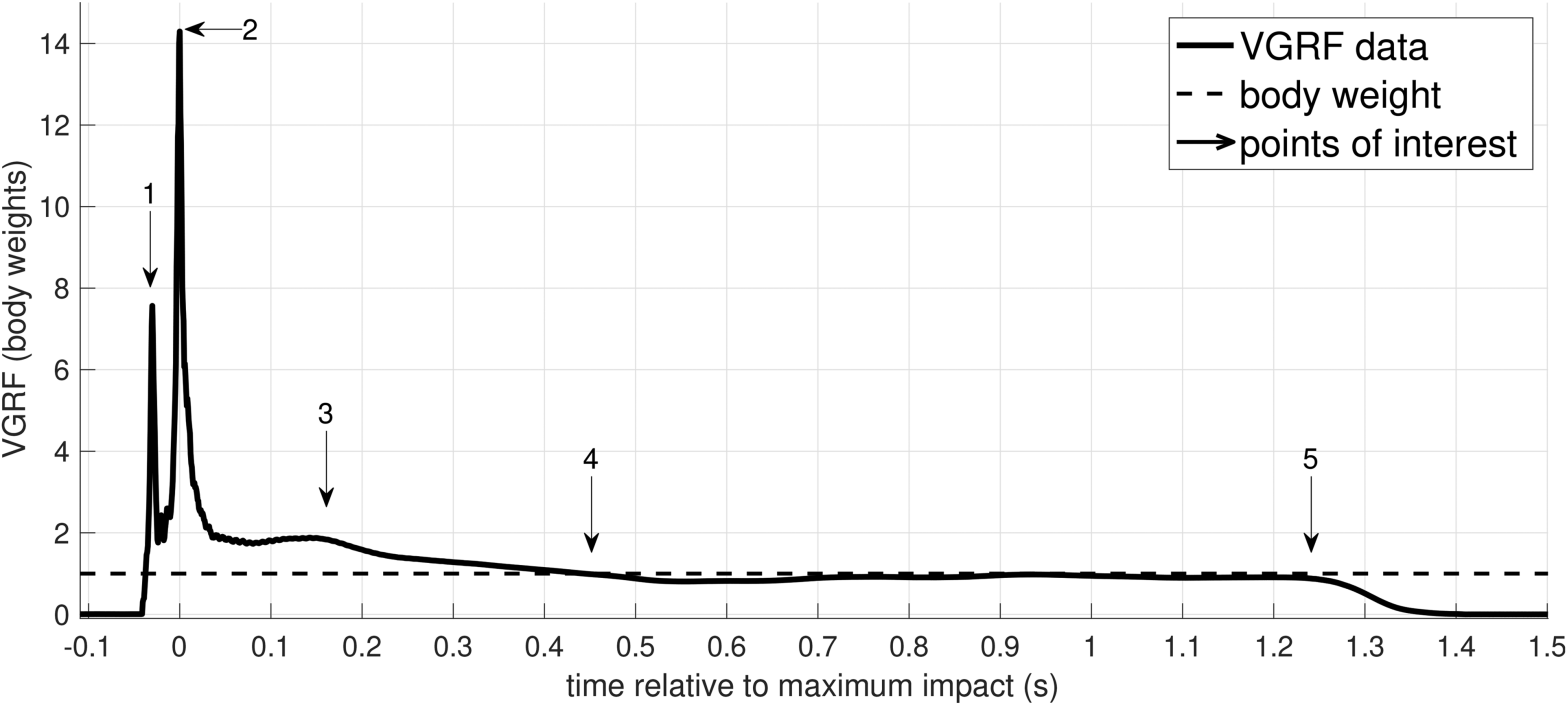
Exemplary measured VGRF-time course during “pop-off” landing. The graph gives a detailed overview of the landing impact, see Figure 1E. Points of interest are (1) toe strike, (2) heel strike, (3) minor peak presumably due to a combination of wobbling masses (24–26) and altered muscle activities (27), (4) reaching of BW, and (5) leaving the force plate.

For this preliminary assessment of VGRF during cheerleading partnerstunts, the flyer-base pairs performed a standard pop-off technique, see picture sequence in Figure 1. The athletes were instructed to perform their routines as if they were training stunts. As the only exception, the teams were prompted to dismount the flyers onto the force plate, while mounting was conducted without constraints. With the flyer standing on their upward, outstretched arms, the bases dipped (bending the knees) both to signal the flyer to dismount and initiate the impetus. Subsequently, upon straightening the knees again, the flyer was thrown in the air, about 20–30 cm additional height. Before landing, the flyer tried to bring her hips forward and grab the wrists of the base. The base ought to decelerate the falling flyer by grabbing her hip thus supporting the landing. A representative impact signal upon landing is shown in Figure 2.

### 2.3 Test protocol and experimental set-up

All measurements were performed over three days in late June and early July 2023, at least two days after a regular training session. Upon arrival, participants were informed about data privacy and the planned stunt measurements and filled out both a case report as well as a detailed Acute Recovery and Stress Scale [ARSS, cf. (28, 29)] questionnaire to measure the psycho-physical effects of the fatiguing workout. The ARSS comprised 32 adjectives, categorized into four recovery items (R1: physical performance, R2: mental performance, R3: emotional balance, and R4: general recovery status) and four stress items (S1: muscular strain, S2: lack of activation, S3: emotional dysbalance, and S4: general stress level). All items were ranked by a seven-point Likert-type scale (ranging from 0 for “strongly disagree” to 6 for “strongly agree”). After an autonomous 10-min warm-up, the athletes were labeled as “rested”. In this state, each athlete performed three counter-movement jumps (CMJ) as well as a sit-and-reach test, both serving as baseline references to asses the effect of the fatiguing workout (23). Negative values for the sit-and-reach test indicate that athletes were not able to reach their toes. The flyer-base pair combinations for the pop-off measurements were chosen by the corresponding author, based on a predetermined succession that ought to ensure a smooth progress and multiple combinations. Due to time restrictions, each flyer did not conduct pop-offs with each base and vice versa. Only few (≤3) measurements of each pair were performed in a row to ensure low latency times for the other athletes. All VGRF measurements were captured by a force plate (KISTLER type: 9287BA, Winterthur, Switzerland) with a 1,500 Hz sampling rate and the software myoForce (Noraxon, USA, version MR3 3.10.30). The bases were placed directly next to the plate with their toes pointing towards the plate but not touching it. The flyers had to plan their landing onto the plate, whereas the bases were instructed not to step on the plate at all. A spotter supported the base during the mounting of the flyer, directing the bases towards the plate, and observing the landing. If the flyer did not land on the plate with both feet or if the base additionally stepped on the plate, the pop-off was labelled “not successful” and left out of the analysis. Out of 197 pop-offs performed in total, 151 were successful (see Table 1).

After the first period of measurements, a 25 min high-intensity workout was performed. The workout comprised two rounds of 18 different exercises, each with 20 repetitions and 10 s pause in between – for example, squats, push-ups, lunges, sit-ups, mountain climbers, or jumping jacks. Athletes thereafter were labeled as “fatigued”. A second set of measurements was conducted, not necessarily in the same order as before. In between the measurements, after approximately 40 min, there was a brief intermission, in which the participants were asked to fill out Borg’s CR10-scale [category-ratio scale from 0 at minimum exertion to 10 at maximum exertion (30), Table 2] as well as a Short Recovery and Stress Scale (SRSS) questionnaire (28, 29). In the SRSS questionnaire, each four adjectives for the eight ARSS items (R1–R4 and S1–S4) are condensed into a single adjective – resulting in a less time-intensive yet well-validated form of the ARSS . The intermission was also used to performed another set of three CMJ and two sit-and-reach tests. For athletes that participated in more than one testing day (see Table 1), the average of all obtained pre-post values were considered in Tables A1 and A2. Pop-off measurements were continued after the intermission, with athletes still labeled as “fatigued”. Note that while bases were active during CMJ, they were passive (catching) during pop-off stunts. From the 151 successful pop-offs, 73 were conducted by rested athletes compared to 78 by fatigued athletes (see Figure 4).

### 2.4 Data analysis and statistics

Post-processing of the data was conducted in MatLab (MathWorks, USA, version R2023b). No filtering procedure was applied to the force-plate data. To ensure comparability, all VGRF-time curves of both pop-off and CMJ were divided by the body weight (BW) of the flyer, shifted to t=0 s at the instant of maximum (peak) impact, and cropped to 100 ms before and 250 ms afterwards, respectively. To test our first hypothesis that fatigued athletes would land (or catch) differently than rested ones, statistical parametric mapping (SPM, i.e. time-dependent, two-sided, paired t-test) was performed using the SPM MatLab package, available at https://spm1d.org (31). Contrary to t-tests for maximum VGRF alone, SPM offers the possibility to detect significant variations along the whole force-time courses. Normality of data was ensured by performing an upstream Kolmogorov–Smirnov goodness-of-fit test. To test our second hypothesis and also undergird the first, a three-way analysis of variance (ANOVA) was carried out, investigating the influence of the factors “base” (levels: B1,…,B8), ”flyer” (levels: F1,…,F7), and “fatigue” (levels: 0 and 1) on the set of peak VGRF values. To test our third hypothesis that landing performance in CMJ and pop-off were related, we calculated the correlation coefficient r for both flyers and bases in both rested and fatigued state. A best-fit line was calculated, whose slope was tested for being significantly different from zero (32) using the test statistic

(1) $$T=\frac{r\cdot \sqrt{n-2}}{\sqrt{1-r^{2}}},$$

where n denotes the number of flyers or bases, respectively. The corresponding p-value was calculated as $p=2\cdot (1-F_{t_{n}}(T))\;,$ where $F_{t_{n}}$ denotes the cumulative distribution function of the t-distribution with n degrees of freedom. Further, an additional ANOVA was carried out to investigate the influence of the aforementioned factors on the quotient between pop-off and CMJ peak VGRF values. To test whether the fatiguing workout had any effect on the sit-and-reach or CMJ performance, as well as on the SRSS, a pre-post paired t-test on the individuals’ mean values was performed. The null hypothesis always assumed that pre- and post-performance were equal with the alternative claiming poorer performances in fatigued athletes – yielding corresponding p-values and Cohen’s d values for measuring effect sizes. As common, the p-value is interpreted as the conditional probability of an absolute pre-post deviation at least as large as the observed one, under the condition that the null hypothesis is true. Regarding the interpretation of Cohen’s d, we follow the “rules of thumb” of Sawilowsky (33), i.e. distinguish between small (d<0.2), medium (0.2≤d<0.8), large (0.8≤d<1.2), very large (1.2≤d<2), and huge (d≥2) effect sizes. Finally, VGRF measurements were additionally equipped with their corresponding time stamp relative to the end of the intense workout. Doing this, we aimed for resolving the individual temporal progress of maximum impact forces.

## 3 Results

We begin with results on the effectiveness of the fatiguing workout. Detailed results of the workout-induced indices are summarized in Appendix A for both, the effects on the athletes’ performances including their CR10 (Borg scale) scores (Table A1) and the result of the ARSS/SRSS questionnaires (Table A2). In brief, the subjective exertion during the workout was rated with a CR10 value of 6.8±1.1 for the flyers and 6.3±1.0 for the bases, i.e. was considered to be “very strong” but not exhaustive throughout. The physical effects of this workout were, on average, the following: (i) a significant decrease in sit-and-reach performance (for all athletes from 11.2±11.5 cm to 9.7±11.5 cm), (ii) a significant decrease in CMJ height (all athletes: from 32.3±7.8 cm to 30.8±7.2 cm), (iii) a slight – but not significant – decrease in CMJ impact force (all athletes: from 4.1±1.3 BW to 3.9±1.0 BW), and (iv) a slight increase in pop-off-impact force (all athletes: from 6.0±1.5 BW to 6.1±1.0 BW). Nevertheless, individual athletes showed opposite trends in certain metrics, see Table A1 for details. The effect sizes of all tests were small (d<0.2) to medium (d<0.5). Contrary, the effect sizes for the stress-recovery-item scores were medium to huge (d>2). As expected, the recovery-item scores (R1–R4) went down, while the stress-item scores (S1–S4) increased. Particularly general stress level (S4), muscular strain (S1), general recovery status (R4), and physical performance (R1) changed (highly) significantly due to the workout. Only non-significant changes occurred for emotional balance (R3) and dysbalance (S3). For detailed values, see Table A2.

**Figure 3:**
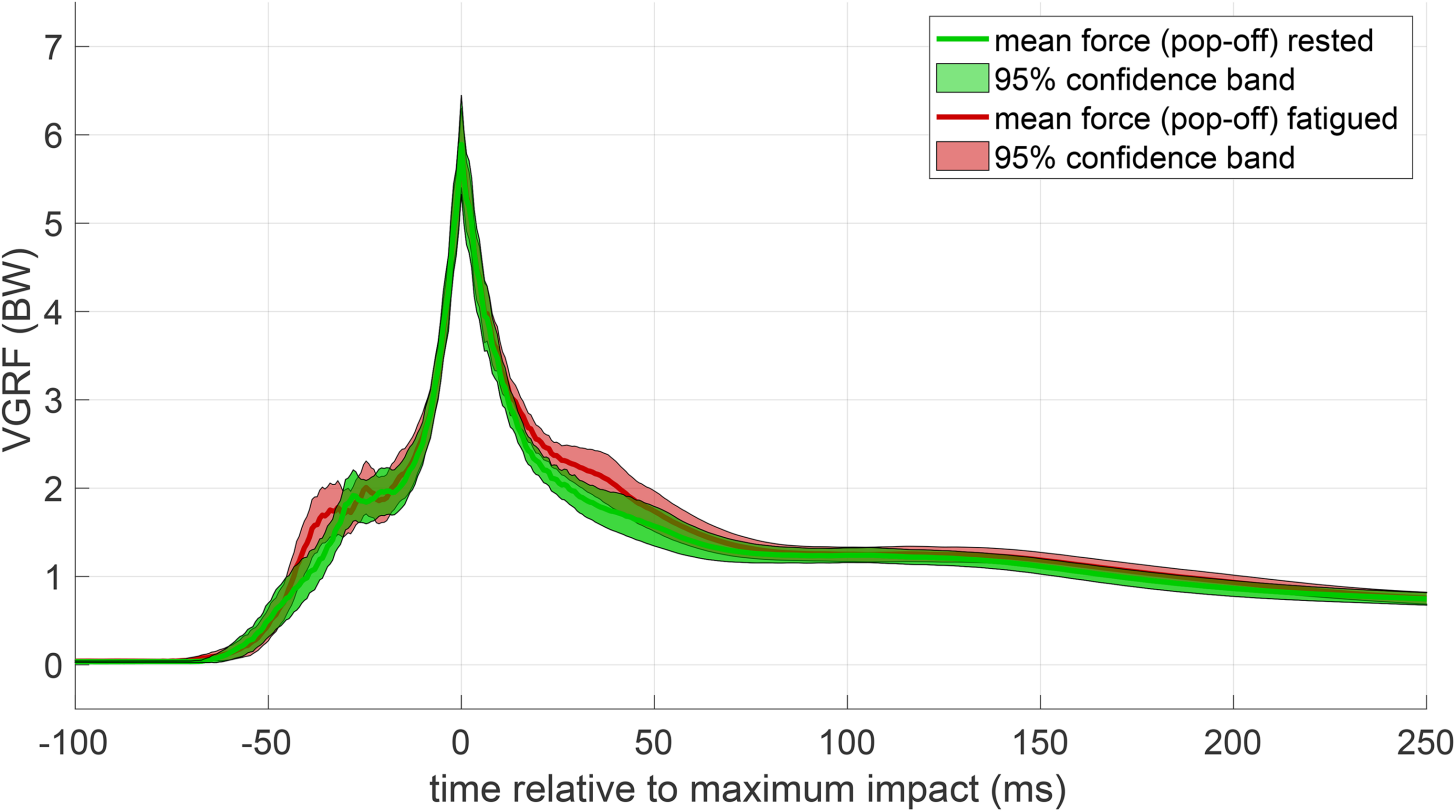
Mean VGRF-time courses of all flyers. Pop-off impact forces before (green) and after (red) the workout are juxtaposed. Lines show the mean force-time course and correspondingly shaded areas the 95% confidence bands.

**Figure B1:**
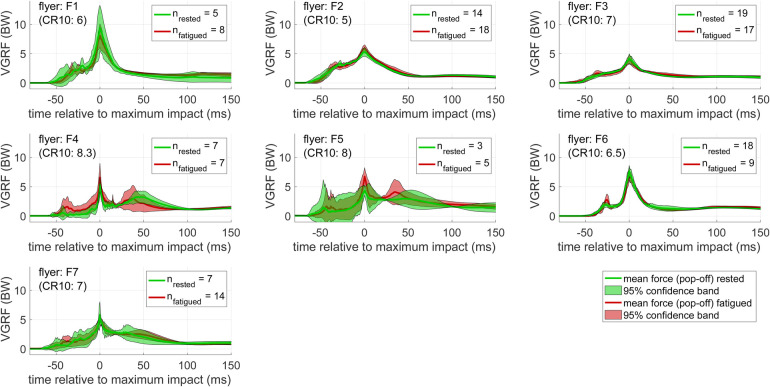
Mean VGRF-time courses of individual flyers (jumping). Pop-off impact forces before (green) and after (red) the workout are juxtaposed. Lines show the mean force-time courses and correspondingly shaded areas the 95% confidence bands. The extent of exhaustion is indicated by alongside CR10 scores (in parentheses) according to Borg’s scale. The quantity of measured pop-offs are given in the legends.

**Figure B2:**
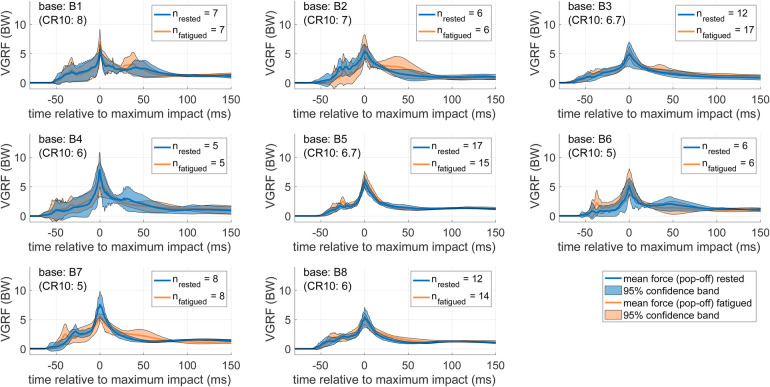
Mean VGRF-time courses of individual bases (catching). Pop-off impact forces before (blue) and after (orange) the workout are juxtaposed. Lines show the mean force-time courses and correspondingly shaded areas the 95% confidence bands. The extent of exhaustion is indicated by alongside CR10 scores (in parentheses) according to Borg’s scale. The quantity of measured pop-offs are given in the legends.

**Figure C1:**
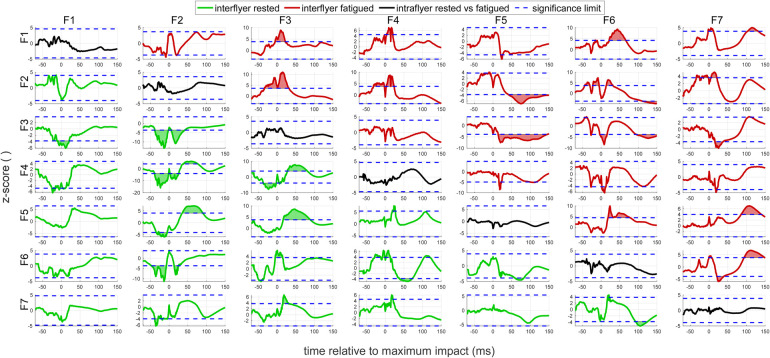
SPM of rested versus fatigued flyers. The subplot in the Nth row and the Mth column shows the result of a statistical parametric mapping of VGRF-time courses from flyer FN versus flyer FM, see Figure B1. If N>M (green curves), the “rested” measurements of flyer FN are tested against those of flyer FM. If N<M (red curves) the “fatigued” measurements are taken instead. If N=M (black curves) the same flyer’s “rested” measurements are tested against the “fatigued” ones. Correspondingly colored shaded areas indicate instances of significant deviation above the significance limit (blue dashed lines).

**Figure C2:**
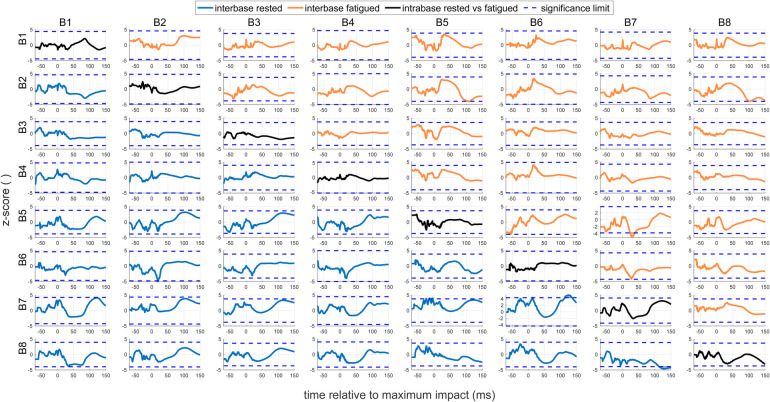
SPM of rested versus fatigued bases. The subplot in the Nth row and the Mth column shows the result of a statistical parametric mapping of VGRF-time courses from bases BN versus flyer BM, see Figure B2. If N>M (blue curves), the “rested” measurements of base BN are tested against those of base BM. If N<M (orange curves) the “fatigued” measurements are taken instead. If N=M (black curves) the same base’s “rested” measurements are tested against the “fatigued” ones. Correspondingly colored shaded areas indicate instances of significant deviation above the significance limit (blue dashed lines).

The effect of the fatiguing workout on the flyers’ landing performances is shown in Figure 3. Contrary to our first hypothesis, the average VGRF-time characteristics are close, except for slightly increased landing forces 30–40 ms before and after the maximum impact. This closeness was also observed at a more detailed resolution on the level of individual flyers and bases before and after the workout (see Figures B1, B2 in Appendix B). Accordingly, a subsequent SPM (see Appendix C) revealed no significant differences at any time instance, neither for the entirety of flyers nor for each flyer individually (diagonal panels in Figure C1). The same held true for the catching performance of the bases (diagonal panels in Figure C2). Consistently, the ANOVA yielded a non-significant (p=0.46) effect of fatigue on peak VGRF values.

**Figure 4:**
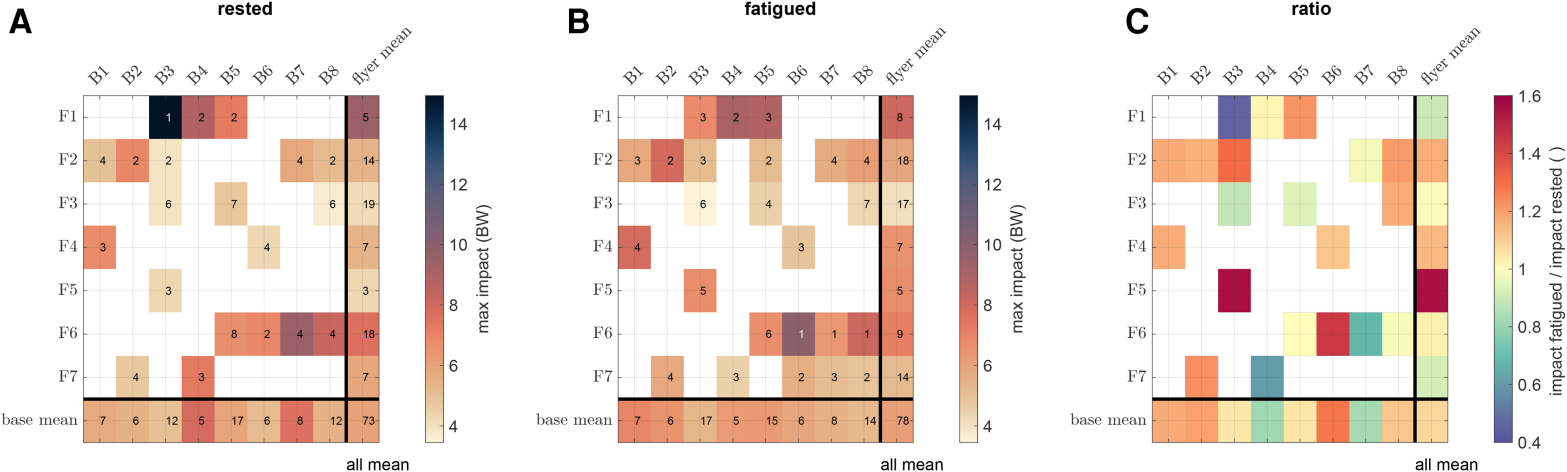
Effect of fatigue on maximum VGRF in individual flyer-base combinations. (**A**) Matrix plot of mean maximum VGRF in rested flyer-base pairs. Colors indicate the magnitude of VGRF. Numbers within the fields represent the corresponding quantity of stunts performed by each pair. (**B**) Matrix plot of mean maximum VGRF in fatigued flyer-base pairs. (**C**) Matrix plot of the ratio between rested and fatigued impact with a divergent colormap indicating an increase (red) or a decrease (blue) in mean maximum VGRF.

To resolve our measurements on an individual performance level, Figure 4 shows the average maximum VGRF values for each flyer-base pair. Note that not every base was paired with every flyer due to the three-day experimental setup and time limitations. Yet, individual pairings seem to have substantial influence on the maximum landing impact. For example, the rested flyers F1 and F6 in Figure 4A showed overall high maximum impact, which however differed by several BWs depending on the catching base. Particularly the flyer-base combination F1-B3 produced a maximum impact of 14.9 BW, despite B3 being the base with the lowest average impact of 4.9 BW of the flyers caught. The smallest single achieved maximum impact was 2.1 BW by the pair F3-B8, who also held the smallest average maximum impact of 3.7 BW.

VGRFs for the fatigued pairs likewise showed a highly individual-dependent profile (Figure 4B). To better assess the changes from before to after the workout, the ratio between rested and fatigued mean maximum VGRF values is shown in Figure 4C. Again contrary to our hypothesis that fatigue would cause higher maximum impact forces (see e.g. pairs F5-B3 and F6-B6), some flyer-base pairs even reduced their impacts by up to 50% (particularly pairs F1-B3, F7-B4, and F6-B7). Across flyers or bases – on average – no significant increase could be discerned. However, an interesting result arose from SPM of inter-flyer and inter-base comparison before and after workout (see lower and upper triangular panels in Figures C1, C2, respectively). Here, the landing characteristics of flyers partly differed significantly around the time instance of maximum impact, particularly for flyer F3. On the other side, the catching characteristics of bases did not differ significantly at any time instance. This was again underpinned by the ANOVA analysis, reporting a highly significant influence of the flyer ($p<10^{-16}$), but only a weak influence of the base (p≈0.086).

**Figure 5:**
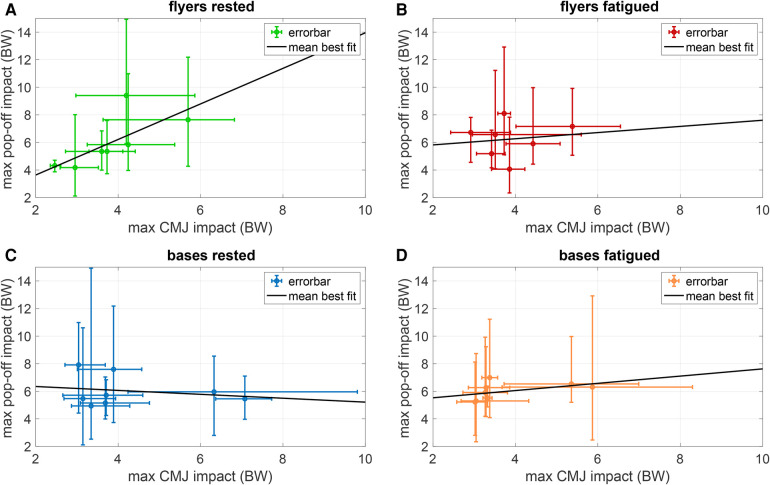
CMJ vs. pop-off impacts in rested and fatigued athletes. Colored dots indicate mean maximum pop-off impact vs. mean max CMJ impact (Table A1) with colors corresponding to VGRF-time curves in Figures B1 and B2. Errorbars indicate the range of the measurements. Best-fitting lines through the mean values are shown in black lines with slopes 1.29 for rested flyers (**A**), 0.22 for fatigued flyers (**B**), −0.14 for rested bases (**C**), and 0.26 for fatigued bases (**D**).

To compare landing impacts with and without partners, maximum VGRF during CMJ and during pop-off measured were juxtaposed in Figure 5. In accordance with our hypothesis that higher landing impacts during CMJ positively relate to higher impact forces during pop-offs, rested flyers showed a high correlation coefficient of r=0.71, which highly significantly differed from zero (p≈0.01), according to Equation (1). However, correlation and thus significance vanished for fatigued flyers (r=0.13, p>0.1). For rested and fatigued bases, both the CMJ and pop-off impacts were expectedly not significantly correlated (|r|<0.4, p>0.1), as their landing mechanics during CMJ have nothing to do with their catching mechanics during pop-offs. When the ANOVA was performed for the quotient between pop-off and CMJ peak impact force, the influence of all factors decreased but did not change the aforementioned significances (flyers: $p<10^{-12}$, bases: p≈0.17, fatigue: p≈0.98).

**Figure 6:**
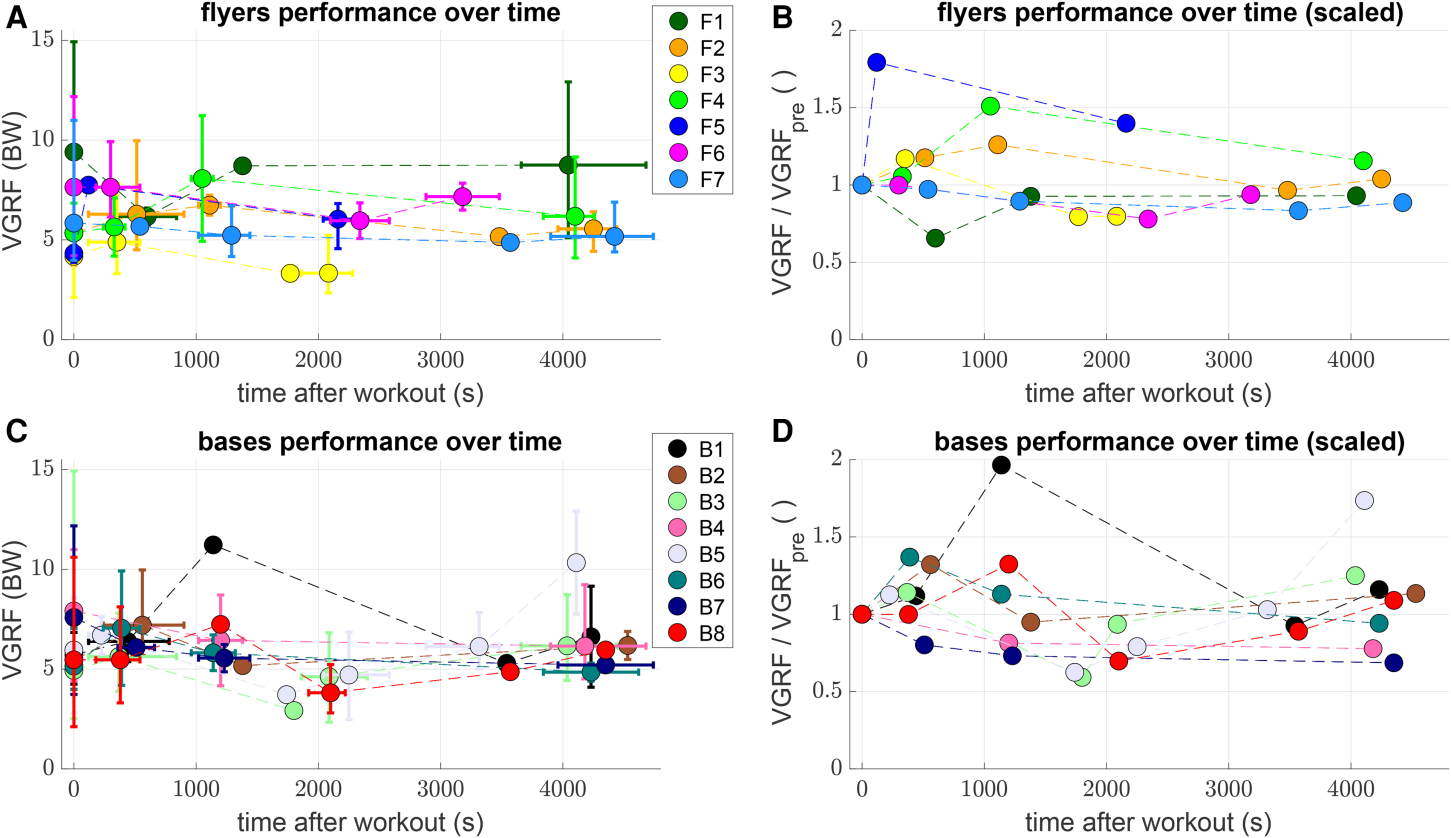
Consider individual changes of impacts over time. Plots of mean maximum VGRF after pop-off vs. mean time after the fatiguing workout at t=0 s (colored dots) in intervals of 15 min (900 s) for both jumping flyers (**A**) and catching bases (**C**). Correspondingly colored errorbars indicate the range, i.e. minimum and maximum value within each interval. (**C**) and (**D**) show the values of (**A**) and (**C**) normalized to the mean VGRF of each individual athlete in the rested state, i.e. the t=0 s value.

As a last observation, the time-resolved VGRF evolution for each flyer and each base are shown in Figure 6. The experiments for rested pairs were hereby lumped into the time instance t=0 s – assumed to serve as a reference – while the time stamps of the pairs after the workout were divided into intervals of 15 min. Within these intervals, the mean time and VGRF for each flyer and base was determined and displayed along with errorbars for the absolute range (Figure 6A,C). To better estimate the changes in performance of each individual, all VGRF values were scaled (additionally to being already normalized to the body weight) to their corresponding rested state value in Figure 6B,D. These plots again suggest a highly individualized response to the fatiguing workout: While some flyers (e.g. F2, F3, F4, and F5) and bases (e.g. B1, B2, B6, and B8) showed an initial increase and a subsequent decrease in VGRF after the workout, some other flyers (e.g. F1 and F7) and bases (e.g. B4 and B7) showed decreased VGRF right after the workout.

## 4 Discussion and implications

### 4.1 Measured impact forces put in context

In this study, we found that maximum VGRF during pop-off landing reached values between 2.1 and 14.9 BW (6.0±1.5 BW for rested and 6.1±1.0 BW for fatigued athletes). The mean to maximum VGRF values are comparable to those reported for somersaults (6.8–13.3 BW) (34) or two-foot landings in gymnastics during training and competition (5–11 BW) (15). The maximum VGRF of 14.9 BW also comes close to the theoretically predicted peak force of 15.3 BW in two-leg landing without support [(35), Equation (4), Figure 3], which we extrapolated by assuming a drop height of 220 cm. On the other hand, the minimum VGRF of 2.1 BW is significantly less than these reported values, which can be explained by the supporting role of the base, see Section 6. It can only be assumed that our measured VGRF values are reliable estimates for typical training environments, as the flyer-base pairs were experienced, but aware of the measurements, and thus keen to perform flawless stunts. Future investigations should also consider real-life training situations, with inexperienced or unwary pairs as well as during unplanned drops.

As to our first hypothesis, our results showed no significant effect of fatiguing on the peak impact forces during pop-offs, which is in line with prior findings on single-leg landing (36, 37). Regarding the second hypothesis, both SPM and ANOVA unambiguously show that the flyer significantly influences the peak impact force, while the base does not. There is, to date, no biomechanical study resolving the issue of flyer-base interaction, see Section 6. The third hypothesis, addressing a possible connection between CMJ and pop-off peak impact forces, could partly be answered by showing a significant correlation in the rested state, while the correlation was not significantly different from zero in the fatigued state. Finally, with regard to monitoring the VGRF profiles over time, we found highly individual patterns with no systematic regularities.

### 4.2 Injuries related to high impacts

Besides injuries caused directly by the measured high impacts (3, 6–9), degenerative changes can occur as a consequence of repetitive overloading (2). To obtain a rough estimate on the stresses acting in the ankle joint, let us consider a mean cartilage area of about 484 mm^2^ [(38), Table 2] in the flyer’s ankles. The mean maximum VGRF (≈6 BW) times the body mean weight of the flyers (Table 1) yields 6.0BW⋅53.0kg/BW⋅9.81N/kg≈3,120N. This force, divided by two feet and the ankle area results in a peak cartilage stress of $3,120\;\text{N}/(2\cdot 484\cdot 10^{-6}\;{\text{m}}^{2})\approx 3.2\;\text{MPa}$ in each ankle. For comparison, maximum stress for flyer F1 (max VGRF 14.9 BW, mass 54.1 kg) lay around 8.2 MPa. Although these values are far below the 15–20 MPa reported as a critical value for causing permanent cell death of the cartilage in the knee (39), the latter lies above the threshold of 4.5 MPa reported as the start of chondrocyte apoptosis (40). Consequently, it could be possible that repetitive stresses after pop-off dismounts lead to long-term degenerative changes of the cartilage, if a 53 kg flyer showed a repetitive maximum impact of more than ≈8 BW. Note here that pop-offs constitute rather simple stunts. Consequently, mean VGRF – and thus injury risk – may increase with or depend on the difficulty of more complex routines.

### 4.3 Landing techniques and training requirements

Based on our rough estimate on ankle stresses during cheerleading stunts and the corresponding injury risk during both cheerleading training and championships, precautions against high impact forces should be taken. Two known precautions include proper landing techniques (41) and suitable surfaces (42).

The former precaution calls for experienced trainers and detailed biomechanical analyses (see also Section 6.). Impact forces occurring during landing cause a considerable increase in external bending moments, particularly in the knee, which requires a proportional increase in muscle forces to counteract these moments (41). Hence, a well-controlled landing phase is crucial – encompassing the management of the center of mass trajectory, body momentum, and angular rotation (15). The posture of the trunk likewise plays an important role in determining landing forces and quadriceps activation. A more flexed upper trunk causes flexed knees and hips, thereby reducing landing forces and thus the associated risk of injury (41, 43). As our results suggest that CMJ peak forces are correlated with pop-off peak forces, and that flyers predominantly influence the latter, we conjecture that improving the CMJ landing in flyers could already have a positive effect on their stunt landing. In this regard, proprioception and strength training may be beneficial for athletes (44). Alternatively, McNair et al. (45) showed that already few verbal instructions, or auditory feedback in general, could help to improve landing techniques, indicated by obtaining significantly lower peak VGRF. Whether these results are transferable to cheerleading yet remains an open question.

The mentioned precautions are subject to local availability. The International Cheer Union (ICU) argues in favor of accessibility, costs, and the grassroot growth of cheerleading to advocate standard foam mat floors instead of spring floors for their championship (46). The European Cheer Union (ECU) follows this recommendation (47), while the CCVD requires spring floors (48) – at least for championships. Aside from these championships, most stunts are being practiced during training sessions at the local clubs. To the best of our knowledge, no reports exist on either prevailing training conditions or injury prevalence in Europe (and thus Germany). Therefore, we are of the opinion that further biomechanical studies on the performance and safety of stunt landings ought to be carried out, e.g. regarding acute or overuse injuries (15, 44) or flooring (42). As a consequence, both ICU and CCVD could establish joint, science-backed guidelines on training conditions within their purview. This holds for planned dismounts, but particularly for accidental drops, which happen frequently during training.

## 5 Limitations

This study has several limitations concerning the quantity and validity of the measurements. For this project, we only evaluated VGRFs of a single stunt (pop-off) in a controlled environment. To the best of our knowledge, we are the first group to report VGRFs during Cheerleading stunts. Hence, no validation of our results with respect to comparable datasets was possible. This holds in particular for the effects of fatiguing training. In this study, we found no significant effect of a high-intensity workout on the athletes’ average VGRF-time performance. However, if the absence of such an effect was caused by insufficient fatigue, extended pauses, poor choice of exercises, or systematic bias in the questionnaire could not be resolved. Future studies will thus have to include a broader spectrum of workouts, stunt techniques, landing surfaces (mats), training methods, and footwear (see Section 4.1) to quantify their corresponding influences reliably (42). Further, kinematic tracking of the flyer (and the base) are necessary to assess differences in dismounting, e.g. variations in dipping, foot placement, landing technique (see Section 6.), or deceleration measures (49). Such kinematic information would also enable to inform inverse dynamic models (26) in order to estimate the force propagation along the flyer’s body. Last, aside from compressive VGRFs, shear forces such as medio-lateral and anterior-posterior GRFs will have to be compared, as these are considered a key factor in landing injuries (50).

## 6 Perspective: the biomechanical role of the base

**Figure 7:**
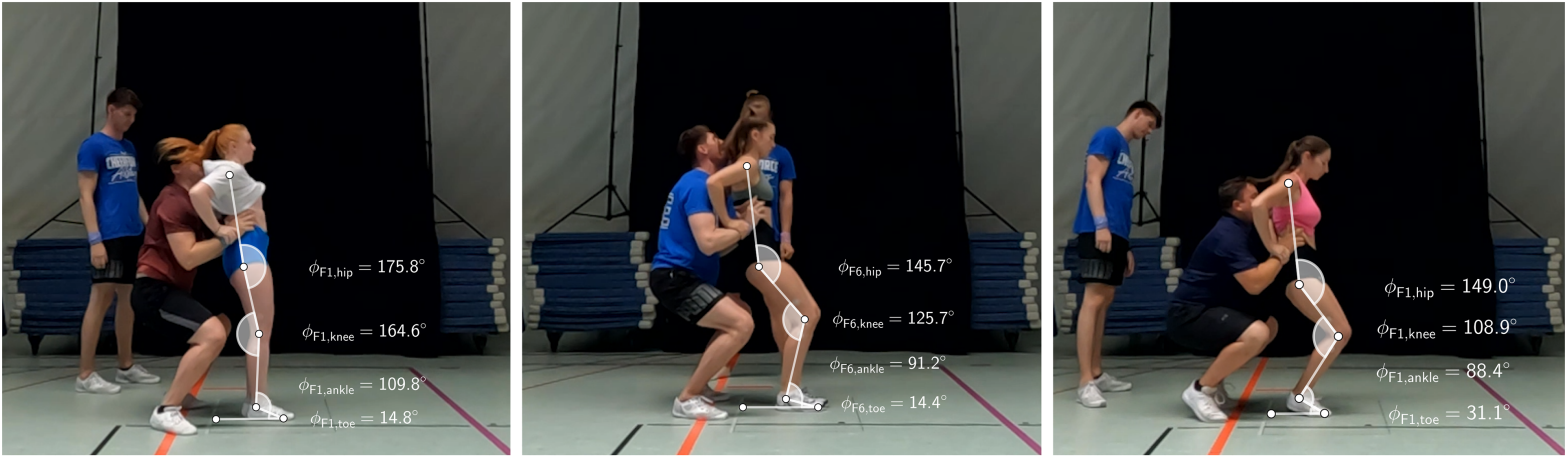
Pose estimation of different flyers during maximum impact. Representative measurements of angles $\phi_{\text{\textbackslash{},flyer},\text{\textbackslash{},joint}}$ for different flyer-base pairs (left: F1-B3, mid: F6-B5, right: F2-B7) and different joints (toe, ankle, knee, and hip) at the time instant of maximum VGRF.

Contrary to usual reports of VGRF in jumping, running, or tumbling, a distinguishing aspect of cheerleading is the existence of a supporting base, see Figures 1 and 7. Depending on the flyer-base interaction, we measured a total range of 12.8 BW (=14.9BW−2.1BW in VGRF during landing from approximately the same height, constituting a vast variability. To our knowledge, no biomechanical study has yet thoroughly investigated the role of supported landing. In this section, we motivate this investigation to be conducted in further studies.

As mentioned in Section 4.3, a variety of heterogeneities already ought to be considered in the flyer’s landing alone. The coupling of the landing with a base adds even more. Here, we have investigated the impacts of rested and fatigued flyer-base partners, but their individual contribution on maximum VGRF could not be resolved. Further, our flyer-base teams were well-attuned to each other, but it can be assumed that the same flyer’s VGRF would differ significantly with a completely unfamiliar base. To what extent, however, has yet to be quantified.

Apart from these individual factors, bases cause a more fundamental change in landing biomechanics. In Figure 7, the differences in body geometry of the flyer at the time instance of maximum impact are shown for three exemplary (rested) flyer-base pairs. For this, screenshots of the videos were manually marked with six points of interest, namely (i) reference point on the floor, (ii) different point on the floor at which the toe joint touched the ground, (iii) estimated mid point of the ankle joint, (iv) estimated mid point of the fibula head at the knee, (v) estimated mid point of the Trochanter major at the hip, (vi) estimated mid point of the shoulder joint. From these markers, we obtained five point-to-point segments (floor, foot, lower leg, upper leg, torso) and thus four joint angles between adjacent segments (toe, ankle, knee, hip). We denoted these angles $\phi_{\text{\textbackslash{},flyer},\text{\textbackslash{},joint}}$ to distinguish flyers and joints. Note that the placement of the markers has not been validated, might be prone to errors, and serves for purely qualitative, descriptive purpose here.

The three exemplary pairs were F1-B3 (Figure 7, left), F6-B5 (mid), and F2-B7 (right) with decreasing maximum VGRFs of 14.9 BW, 7.5 BW, and 3.7 BW, respectively. Whether the effects stated hereinafter were statistically significant, or how the geometric changes propagated through the entire landing phase, was not further investigated due to low image quality and lack of clearly visible body landmarks. Solely based on these three samples, we note the following observations. First, the hip joint marker in all three athletes is placed more dorsally than the ankle joint marker. In gymnasts’ landing, this would certainly result in toppling down backwards, as the center of mass is not supported by the feet. Second, the knee joint markers were positioned further forward the lower the maximum VGRF. For flyer F2, the knee joint even protruded over the toes. Third, in accordance with Blackburn and Padua (41), a more backwards extended trunk seemed to correspond to higher VGRF. Fourth, flyer F2 was able to distinctly reduce the knee angle ($\phi_{F2,\mathrm{knee}}=113.4^{\circ }$) in comparison to flyer F6 ($\phi_{F6,\mathrm{knee}}=125.7^{\circ }$), by not landing on the heel, but on the toes only, thereby increasing the toe joint angle.

In summary, further biomechanical studies on cheerleader’s landing ought to consider these geometric alterations of the flyers in order to provide insight on how to reduce the risk of injuries.

## Data Availability

The raw data supporting the conclusions of this article will be made available by the authors, without undue reservation.

## Appendix

### A. Stress-recovery data

**Table A1:** Effects of the fatiguing workout on athletes.

Identifier	CR10 score	Sit and reach		CMJ height		CMJ impact		pop-off impact	
	( )	(cm)		(cm)		(BW)		(BW)	
		Pre	Post	Pre	Post	Pre	Post	Pre	Post
F1	6.0	16.2	13.0	29.5	26.4	4.2	3.7	9.4	8.1
F2	5.0	20.6	19.7	25.9	21.4	3.7	4.4	5.3	5.9
F3	7.0	17.0	15.6	29.4	31.5	3.0	3.9	4.2	4.1
F4	8.3	19.2	18.5	25.9	26.7	3.6	3.5	5.3	6.6
F5	8.0	19.0	16.6	25.2	25.0	2.5	2.9	4.3	6.7
F6	6.5	19.0	18.7	31.3	31.2	5.7	5.4	7.6	7.2
F7	7.0	24.3	23.7	28.5	25.2	4.2	3.5	5.8	5.2
All flyers	6.8	19.3	18.0	28.0	26.8	3.8	3.9	6.0	6.2
	± 1.1	± 2.6	± 3.9	± 2.3	± 3.6	± 1.0	± 0.8	± 1.9	± 1.3
		p=0.02		p=0.25		p=0.84		p=0.64	
		d=0.45		d=0.39		d=0.05		d=0.14	
B1	8.0	2.7	1.1	27.1	26.0	3.7	3.4	5.7	7.0
B2	7.0	18.7	14.9	46.3	43.8	7.1	5.4	5.5	6.5
B3	6.7	-3.8	-3.8	42.3	41.4	3.3	3.0	4.9	5.3
B4	6.0	19.1	15.2	39.0	37.3	3.0	3.3	7.9	6.3
B5	6.7	12.0	14.1	46.7	42.6	6.3	5.9	6.0	6.3
B6	5.0	-5.4	-11.4	33.7	29.2	3.7	3.3	5.2	5.9
B7	5.0	-12.8	-11.9	21.4	23.5	3.9	3.3	7.6	5.5
B8	6.0	2.6	1.7	32.5	31.1	3.1	3.0	5.5	5.2
All bases	6.3	4.1	2.5	36.1	34.4	4.28	3.8	6.0	6.0
	± 1.0	± 11.6	± 11.3	± 9.1	± 7.9	± 1.53	± 1.1	± 1.1	± 0.6
		p=0.13		p=0.05		p=0.05		p=0.97	
		d=0.14		d=0.21		d=0.34		d=0.02	
All athletes	6.5	11.2	9.7	32.3	30.8	4.1	3.9	6.0	6.1
	± 1.1	± 11.5	± 11.5	± 7.8	± 7.2	± 1.3	± 1.0	± 1.5	± 1.0
		p=0.01		p=0.02		p=0.20		p=0.76	
		d=0.13		d=0.19		d=0.20		d=0.08	

Different measures before and after the intense workout are summarized. The CR10 score of Borg’s psychophysical exertion scale (0: minimum, 10: maximum), the sit-and-reach range, CMJ height and impact, as well as pop-off impact are thought to indicate the strenuousness of the workout. Statistical measures such as mean, standard deviation (± sign), as well as p-values and Cohen’s d effect sizes of the paired t-tests are given for all flyers, all bases, and all athletes, respectively.

**Table A2:** Results from recovery-stress questionnaires.

	R1		R2		R3		R4		S1		S2		S3		S4	
Identifier	Pre	Post	Pre	Post	Pre	Post	Pre	Post	Pre	Post	Pre	Post	Pre	Post	Pre	Post
F1	2.5	2	4	2	4.25	2	2	0	2.25	4	0.25	2	1.5	0	3.75	5
F2	4.5	4	4.5	4	5.25	5	4.5	2	0.25	4	0.5	1	0.5	0	0.75	4
F3	5.25	3	5.25	3	5.75	5	5	2	0.25	4	0	1	0.25	2	0.5	4
F4	3.75	3	3.75	3.33	4.1	3.66	3.75	3	1.7	4.7	1.6	2.3	2	2	1.8	4.7
F5	2.5	2	3.5	4	3.25	3	2	3	5.5	3	2	3	3.25	2	4	5
F6	4.75	3.5	4.5	4.5	4.9	5	3.25	2.5	3.6	4	0.6	2.5	1.5	1	2.9	3
F7	3.25	3	5	5	4.5	6	2.5	2	3	4	1.75	1	1.25	2	3.5	4
All flyers	3.79	2.93	4.36	3.69	4.57	4.24	3.29	2.07	2.36	3.96	0.96	1.83	1.46	1.29	2.46	4.24
	± 1.01	± 0.68	± 0.6	± 0.93	± 0.76	± 1.29	± 1.11	± 0.94	± 1.74	± 0.46	± 0.74	± 0.77	± 0.92	± 0.88	± 1.34	± 0.66
	p=0.02		p=0.14		p=0.46		p=0.06		p=0.11		p=0.04		p=0.69		p=0.01	
	d=0.92		d=0.79		d=0.29		d=1.10		d=1.16		d=1.07		d=0.18		d=1.57	
B1	4.25	0	5.25	1	5.5	0	4.25	1	1.25	6	0.75	2	2.25	4	3	5
B2	4.25	2	5.5	4	5.25	4	4	1	1.5	3	0.5	1	0.25	1	1	4
B3	5.1	1.7	5.3	4.3	5.3	4	5	2.3	2.7	3.7	0	1.7	0	1.3	0.75	4.3
B4	4	2	5	2	3.5	4	4.5	2	0.75	4	1.75	1	1	1	1	5
B5	3.2	1.7	4.5	3	4.1	4	3.1	1.7	0.9	3.7	1.1	2.7	1.75	1	2.9	4.3
B6	5	4	6	5	5.25	6	5.25	4	2.25	4	0	0	0.25	0	0.25	2
B7	5	3	4.5	5	5	5	3.25	2	2.5	4	0.25	1	1	0	1.25	4
B8	4.25	2	4.6	3	4.6	4.5	3.6	2	2.6	3.5	2.1	2	2.4	1.5	1.9	4
All bases	4.38	2.05	5.08	3.41	4.81	3.94	4.12	2.0	1.81	3.99	1.37	1.43	1.11	1.23	1.51	4.08
	± 0.6	± 1.07	± 0.5	± 1.34	± 0.65	± 1.63	± 0.73	± 0.88	± 0.75	± 0.82	± 1.55	± 0.79	± 0.87	± 1.17	± 0.94	± 0.88
	p≪0.01		p=0.01		p=0.26		p≪0.01		p≪0.01		p=0.09		p=0.77		p≪0.01	
	d=2.51		d=1.54		d=0.66		d=2.45		d=2.60		d=0.76		d=0.10		d=2.65	
All athletes	4.1	2.46	4.74	3.54	4.7	4.08	3.73	2.03	2.07	3.97	1.18	1.61	1.28	1.25	1.95	4.15
	± 0.87	± 1.01	± 0.66	± 1.17	± 0.71	± 1.49	± 1.01	± 0.91	± 1.34	± 0.68	± 1.26	± 0.8	± 0.91	± 1.04	± 1.24	± 0.79
	p≪0.01		p<0.01		p=0.16		p≪0.01		p≪0.01		p<0.01		p=0.93		p≪0.01	
	d=1.69		d=1.22		d=0.52		d=1.70		d=1.74		d=0.92		d=0.02		d=2.05	

Juxtaposing the four recovery items (R1: physical performance, R2: mental performance, R3: emotional balance, and R4: general recovery status) and the four stress items (S1: muscular strain, S2: lack of activation, S3: emotional dysbalance, and S4: general stress level) before (pre) and after (post) the fatiguing workout. Statistical measures such as mean, standard deviation (± sign), as well as p-values and Cohen’s d effect sizes of the paired t-tests are given for all flyers, all bases, and all athletes, respectively. If 0.001<p<0.01 it is indicated by p<0.01, if p≤0.001 we write p≪0.01.
